# 

**Published:** 1890-02

**Authors:** 


Entered at the Post Office at Austin, Texas, as Second-class Mail Matter.

Vol. V. FEBRUARY, 1890. No. 8.

### DANIEL'S MEDICAL JOURNAL

Subscription, $2.00 a Year, in Advance — Single Copies, Twenty-five Cents.

F. E. DANIEL, M. D., Editor and Publisher, AUSTIN, TEXAS.

Eugene Von Boeckmann, Steam Book and Job Printer, Austin, Texas.

